# Biomechanical Analysis of the Human Finger Extensor Mechanism during Isometric Pressing

**DOI:** 10.1371/journal.pone.0094533

**Published:** 2014-04-14

**Authors:** Dan Hu, David Howard, Lei Ren

**Affiliations:** 1 School of Mechanical, Aerospace and Civil Engineering, University of Manchester, Manchester, United Kingdom; 2 School of Computing, Science and Engineering, University of Salford, Manchester, United Kingdom; 3 State Key Laboratory of Automotive Simulation and Control, Jilin University, Changchun, P.R. China; University of South Australia, Australia

## Abstract

This study investigated the effects of the finger extensor mechanism on the bone-to-bone contact forces at the interphalangeal and metacarpal joints and also on the forces in the intrinsic and extrinsic muscles during finger pressing. This was done with finger postures ranging from very flexed to fully extended. The role of the finger extensor mechanism was investigated by using two alternative finger models, one which omitted the extensor mechanism and another which included it. A six-camera three-dimensional motion analysis system was used to capture the finger posture during maximum voluntary isometric pressing. The fingertip loads were recorded simultaneously using a force plate system. Two three-dimensional biomechanical finger models, a minimal model without extensor mechanism and a full model with extensor mechanism (tendon network), were used to calculate the joint bone-to-bone contact forces and the extrinsic and intrinsic muscle forces. If the full model is assumed to be realistic, then the results suggest some useful biomechanical advantages provided by the tendon network of the extensor mechanism. It was found that the forces in the intrinsic muscles (interosseus group and lumbrical) are significantly reduced by 22% to 61% due to the action of the extensor mechanism, with the greatest reductions in more flexed postures. The bone-to-bone contact force at the MCP joint is reduced by 10% to 41%. This suggests that the extensor mechanism may help to reduce the risk of injury at the finger joints and also to moderate the forces in intrinsic muscles. These apparent biomechanical advantages may be a result of the extensor mechanism's distinctive interconnected fibrous structure, through which the contraction of the intrinsic muscles as flexors of the MCP joint can generate extensions at the DIP and PIP joints.

## Introduction

The structural and functional complexities of the human finger have long been recognised [1]–[5]. Effective function of the finger requires precise coordination of multiple muscles and the resulting finger motion is constrained by the forces exerted by the joint capsules, ligaments and joint articular surfaces. In manual activities, the highly complex musculoskeletal system of the hand and forearm is well coordinated to generate appropriate fingertip forces and finger postures. A good understanding of the biomechanical mechanisms of the finger would not only improve our knowledge of normal finger function and the etiology of hand diseases, but may also significantly improve prosthetic and biomimetic hand design.

**Figure 1:**
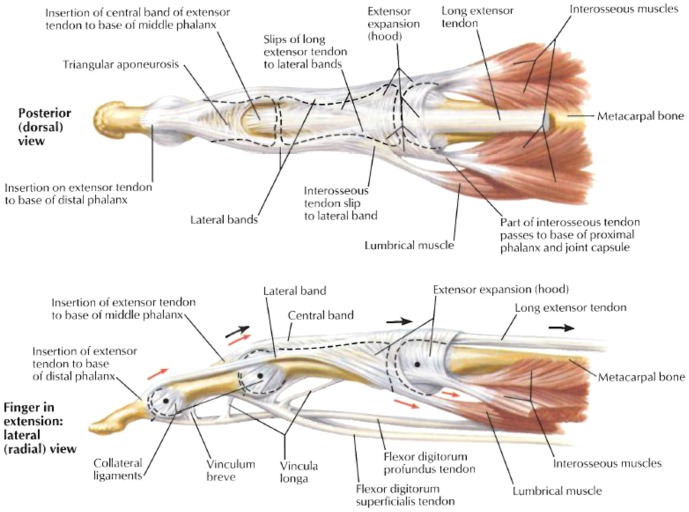
Musculotendonal structure of the human finger. The musculotendonal structure of the human finger from posterior (dorsal) and lateral (radial) views (from Netter, 2002)

However, finger mechanics is complicated by the finger extensor mechanism (also referred to as the extensor apparatus, extensor assembly or extensor expansion), which is a complex tendon network that brings together the forces of the lumbrical, interossei, and long extensor to produce precise functional movements of the phalanxes (see Figure 1). In recent decades, a number of studies have been conducted to investigate its anatomical structure [6]–[16] and the spatial relationships between its different components, to quantify its geometric configuration [17] and material properties [18]. In addition, recently there has been increasing use of extensor mechanism models for the biomechanical analysis of finger function [19]–[27]. However, despite this, little is known about how the extensor mechanism affects the mechanical loadings at finger joints and muscles.

Therefore, in this study, we aim to investigate the biomechanical effect of the extensor mechanism (tendon network) during isometric pressing using a combined experimental and modelling approach. Fingertip force and finger posture were recorded using a force plate and a three-dimensional (3D) motion analysis system. Force analysis was conducted using two different finger models, a minimal model excluding the extensor mechanism and a full model including the extensor mechanism. In this way, the effects of this complex tendon network on finger joint contact forces and extrinsic and intrinsic muscle forces were analysed. However, it should be noted that the conclusions drawn are based on interpreting the differences between the results generated by the two models and, as such, cannot be quoted with the confidence one would associate with wholly experimental results.

## Methods

### Notation

*PF*: primary flexor

*PE*: primary extensor

*FDP*: flexor digitorum profundus

*FDS*: flexor digitorum superficials

*TE*: terminal extensor

*ES*: extensor slip

*LE*: long extensor

*RI*: radial interosseous

*UI*: ulnar interosseous

*LU*: lumbrical

*RB*: radial band

*UB*: ulnar band

*DIP*: distal interphalangeal

*PIP*: proximal interphalangeal

*MCP*: metacarpophalangeal

*a_PF_DIP_FL,_ a_PF_PIP_FL,_ a_PF_MCP_FL_*: flexion/extension moment arm of *PF* around DIP, PIP and MCP joint

*a_PE_DIP_FL,_ a_PE_PIP_FL,_ a_PE_MCP_FL_*: flexion/extension moment arm of *PE* around DIP, PIP and MCP joint

*a_RI_MCP_FL,_ a_UI_MCP_FL_*: flexion/extension moment arm of *RI* and *UI* around MCP joint

*a_RI_MCP_AD,_ a_UI_MCP_AD_*: adduction/abduction moment arm of *RI* and *UI* around MCP joint

*a_TE_DIP_FL_*: flexion/extension moment arm of *TE* around DIP joint

*a_FDP_DIP_FL,_ a_FDP_PIP_FL,_ a_FDP_MCP_FL_*: flexion/extension moment arm of *FDP* around DIP, PIP and MCP joint

*a_ES_PIP_FL,_ a_UB_PIP_FL,_ a_RB_PIP_FL_*: flexion/extension moment arm of *ES*, *UB* and *RB* around PIP joint

*a_LE_MCP_FL,_ a_RI_MCP_FL,_ a_UI_MCP_FL,_ a_LU_MCP_FL_*: flexion/extension moment arm of *LE*, *RI*, *UI* and *LU* around MCP joint

*a_RI_MCP_AD,_ a_UI_MCP_AD,_ a_LU_MCP_AD_*: adduction/abduction moment arm of *RI*, *UI* and *LU* around MCP joint

*θ*_1_,*θ*_2_,*θ*_3_,*θ*_4_: angles between phalange segments and X axis of global coordinate system (which is horizontal)

*θ_PF_DIP,_ θ_PF_PIP,_ θ_PF_MCP_*: angle between *PF* and X axis of global coordinate system at DIP,PIP and MCP joint

*θ_PE_DIP,_ θ_PE_PIP,_ θ_PE_MCP_*: angle between *PE* and X axis of global coordinate system at DIP, PIP and MCP joint

*θ_x_RI_MCP,_ θ_y_RI_MCP,_ θ_z_RI_MCP_*: angles between *RI* and the X,Y,Z axes of the global coordinate system at MCP joint

*θ_x_UI_MCP,_ θ_y_UI_MCP,_ θ_z_UI_MCP_*: angles between *UI* and the X,Y,Z axes of the global coordinate system at MCP joint

*θ_FDP_DIP,_ θ_FDP_PIP,_ θ_FDP_MCP_*: angle between *FDP* and X axis of global coordinate system at DIP, PIP and MCP joint

*θ_TE_DIP_*: angle between *TE* and X axis of global coordinate system at DIP joint

*θ_ES_PIP_*: angle between *ES* and X axis of global coordinate system at PIP joint

*θ_LE_MCP_*: angle between *LE* and X axis of global coordinate system at MCP joint

*θ_x_UB_PIP,_ θ_y_UB_PIP,_ θ_z_UB_PIP_*: angles between *UB* and the X,Y,Z axes of the global coordinate system at PIP joint

*θ_x_RB_PIP,_ θ_y_RB_PIP,_ θ_z_RB_PIP_*: angles between *RB* and the X,Y,Z axes of the global coordinate system at PIP joint

*θ_x_LU_MCP,_ θ_y_LU_MCP,_ θ_z_LU_MCP_*: angles between *LU* and the X,Y,Z axes of the global coordinate system at MCP joint

*l*_1_, *l*_2_, *l*_3_: phalangeal lengths

*P_x_*, *P_y_*, *P_z_*: measured fingertip forces

### Ethics Statement

This study was approved by Manchester University's Institutional Review Board, and the subjects provided written informed consent to participate in the experimental work.

### Static pressing measurements

**Figure 2:**
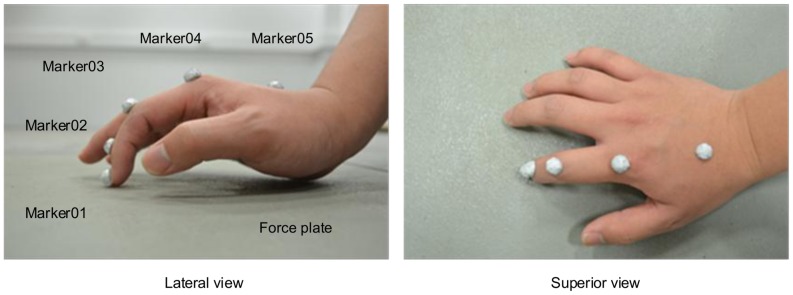
Experimental setup. Experimental setup for the measurement of 3D fingertip force and finger posture during maximum voluntary isometric pressing. The subjects' wrists were not touching the surface of the force plate while measurements were being conducted.

**Figure 3:**
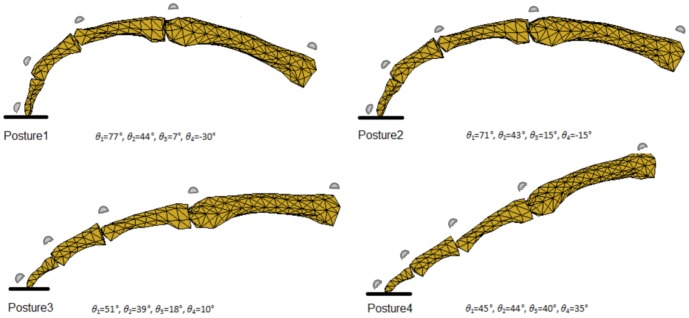
The four finger pressing postures. The four pressing postures, varying from flexed to fully extended, used in the experimental work. The segmental angles (*θ*_1_, *θ*_2_, *θ*_3_, *θ*_4_) are defined in Figure 4.

The experimental work involved six male subjects (age: 26±1years, weight: 75.8±8.1 kg, height: 174±4 cm) recruited from the University's population of postgraduate students. The subjects were instructed to press the force plate surface using their index finger for approximately 3 seconds using maximum voluntary isometric force (see Figure 2), while other parts of the body were not allowed to touch the force plate. Four different finger postures were adopted during static pressing, ranging from very flexed to fully extended (see Figure 3). Each experimental condition was measured ten times. Motion data were recorded at 200 Hz using a six-camera motion analysis system (Vicon, Oxford, UK) and the 3D external force acting on the fingertip was recorded at 1000 Hz using a force plate (Kistler, Switzerland). Referring to Figure 3, to capture finger motion, five semi-reflective markers of 8 mm diameter were attached to the distal phalange dorsal head (Marker01), middle phalange dorsal head (Marker02), proximal phalange dorsal head (Marker03), metacarpal bone dorsal head (Marker04), and metacarpal bone dorsal base (Marker05).

The raw marker data were processed using bespoke programs written in Matlab (Mathworks, MA, USA). All trials with more than 10 consecutive missing frames were discarded. After fill-gap processing, the data were filtered using a low-pass zero-lag fourth-order Butterworth digital filter with a cut-off frequency of 6.0 Hz. For both marker and force plate records, only the data in the middle of the trials was used when the subject had reached a steady isometric pressing condition. After data processing, the measured 3D external fingertip load *P* (*P_x_*, *P_y_*, *P_z_*) and phalange angles (*θ*_1_,*θ*_2_,*θ*_3_,*θ*_4_) at a representative instant in time were used for the following biomechanical force analyses.

### Minimal model without extensor mechanism

**Figure 4:**
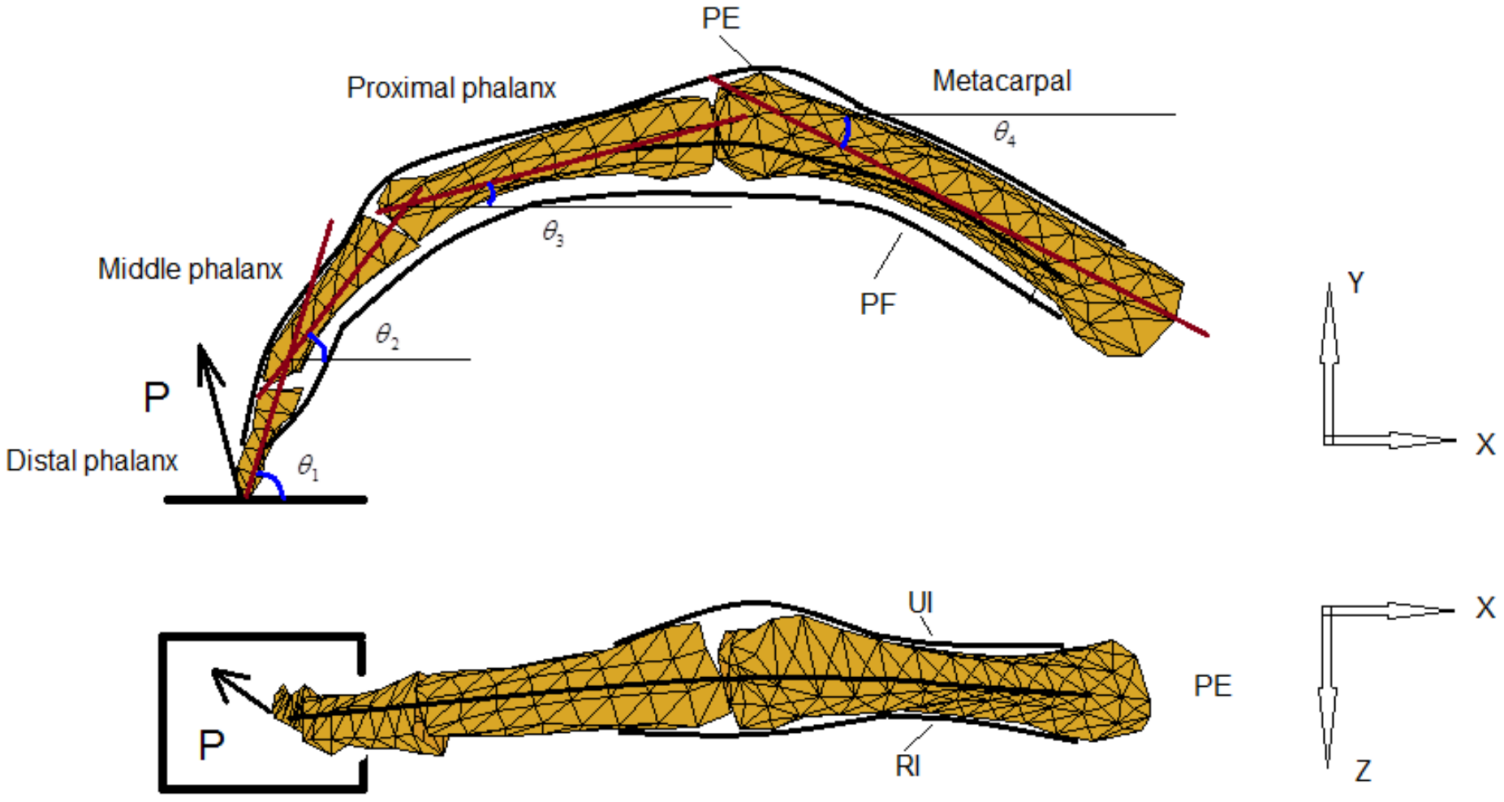
Minimal Model of the finger without extensor mechanism. Posterior (dorsal) and lateral (radial) views of the Minimal Model of the index finger without extensor mechanism. Four equivalent muscles (*PF*, *PE*, *UI*, *RI*) are considered to represent the actions of the finger flexor, extensor, lateral ulnar and lateral radial muscle groups respectively.



To represent the index finger musculoskeletal structure without the extensor mechanism, a simple 3D multi-segment model was constructed by scaling a standard finger model provided in the OpenSim biomechanical simulation environment [28]. The geometry of the digital bones was extracted from the OpenSim software and all other geometry (e.g. muscle insertion, origin positions etc.) was defined by referring to the Primal Pictures 3D anatomical software (Primal Picture Ltd., London, UK) and the literature [12]. The model consists of four segments, namely the distal, middle and proximal phalanxes, and the metacarpal bone, and three joints, namely the DIP, PIP and MCP. Both the DIP and PIP were modelled as hinge joints, each with 1 degree of freedom (DoF), and the MCP was modelled as a saddle joint with 2 DoF (see Figure 4). For this 4-DoF multi-segment system, a minimum of four muscles are needed to balance the external load during static pressing. Referring to Figure 4, a primary extensor (*PE*) was included to represent the combined action of the extensor muscles (mainly the long extensor) spanning the three joints. A primary flexor (*PF*) was used to represent the action of the flexor muscles (mainly the *FDP* and *FDS*). Two lateral muscles (*UI* and *RI*) are included on each side of the finger. This is analysed as a statically determinate system at equilibrium with the required minimum number of muscles. The force and moment equilibrium equations were derived as follows for each of the three joints (DIP, PIP and MCP respectively)
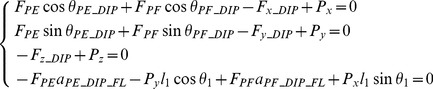
(1)


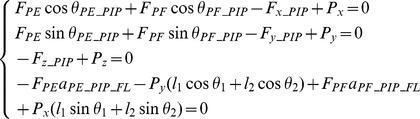
(2)




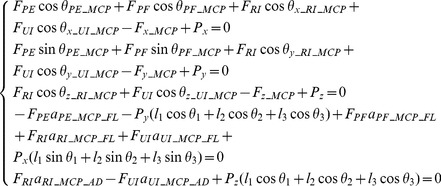
(3)


Where the various muscle and tendon forces (*F*_identifier_), moment arms (*a*_identifier_), angles (*θ*_identifier_), and segment lengths (*l*_identifier_) are defined in the notation list.

Equations 1 to 3 result in a total of 13 equilibrium equations with 13 unknowns (4 muscle forces and 9 bone-to-bone contact forces at the 3 joints). Therefore, the system is statically determinate and all of the unknowns can be determined from the measured finger posture and fingertip load during static pressing.

### Full model with extensor mechanism

**Figure 5:**
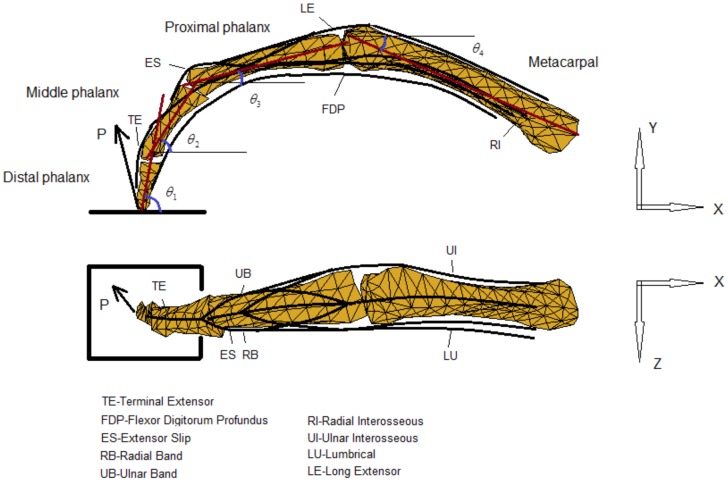
Full Model of the finger with extensor mechanism. Posterior (dorsal) and lateral (radial) views of the Full Model of the index finger with extensor mechanism (tendon network). In addition to the finger extensor muscle LE and flexor muscle FDP, the three major intrinsic muscles (*UI*, *RI* and *LU*) are included.



To investigate the effect of the extensor mechanism, a second multi-segment finger model was developed that represents the extensor apparatus as an interconnected tendon network (see Figure 5). The model shares the same segments, joint configurations, and bone geometry as the minimal model but with additional muscles and tendons. Referring to Figure 5, the five muscles included are the *LE*, *FDP*, *RI*, *UI* and *LU*. As the major extensor, *LE* has a similar function to that of the *PE* muscle in the minimal model. As the major flexor, *FDP* has a similar function to that of the *PF* muscle in the minimal model. In order to represent the key structural features of the extensor mechanism, another muscle (*LU*) is added to the full model on the radial side in addition to the *RI* and *UI* muscles. The force and moment equilibrium equations were derived as follows for each of the three joints (DIP, PIP and MCP respectively).
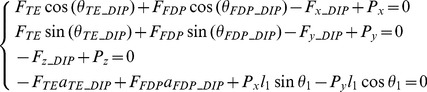
(4)


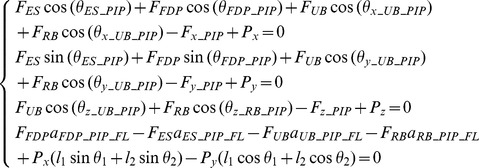
(5)




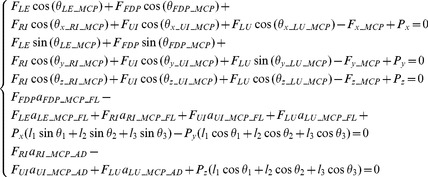
(6)


Where the various muscle and tendon forces (*F*_identifier_), moment arms (*a*_identifier_), angles (*θ*_identifier_), and segment lengths (*l*_identifier_) are defined in the notation list.

Equations 4-6 define a statically indeterminate system at equilibrium with 13 equations and 18 unknowns. To resolve the static indeterminacy problem, the equations below are included, which are based on previous anatomical studies and cadaveric testing [29]. These equations describe the empirical distribution of forces between the muscles (*F_RI_*, *F_UI_*, *F_LU_*, *F_LE_*) and the tendon components (*F_RB_*, *F_UB_*, *F_TE_*, *F_ES_*) of the extensor mechanism [30], [31]. 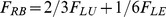
(7)




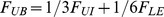
(8)




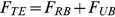
(9)




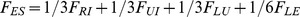
(10)




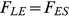
(11)


A more sophisticated optimisation based method could be employed to improve the solution of this statically indeterminate system [35]–[38]. However, finding an appropriate optimisation criterion may be challenging. Equations 4–11 can be used to solve for the bone-to-bone contact forces at all three joints and also the forces within the musculotendon network of the extensor mechanism for each measured finger posture and fingertip force.

### Statistical analysis

All statistical analyses were conducted using SPSS 20.0 software (IBM, Armonk, NewYork, USA). The effects of finger model and posture on joint bone-to-bone contact forces and muscle forces were analysed using analysis of variance (ANOVA) with repeated measurements using a linear mixed model approach taking into account intra- and inter-subject variability. The different finger models and postures were the fixed effects, and subjects and trials were random effects. Differences between the two models and between each pair of postures were tested using Fisher's least significant difference (LSD) multiple comparison based on the least-squared means.

## Results

For all subjects, the measured finger joint angles (*θ*_1_,*θ*_2_,*θ*_3_,*θ*_4_) and fingertip forces (*P_x_*, *P_y_*, *P_z_*) for each static pressing trial were used as inputs to both the minimal model and the full model. These models were implemented using bespoke programs written in Matlab (Mathworks, MA, USA). In this way, biomechanical analyses were conducted to assess the bone-to-bone contact forces at each joint and also the forces in the muscles and tendon components.

**Figure 6:**
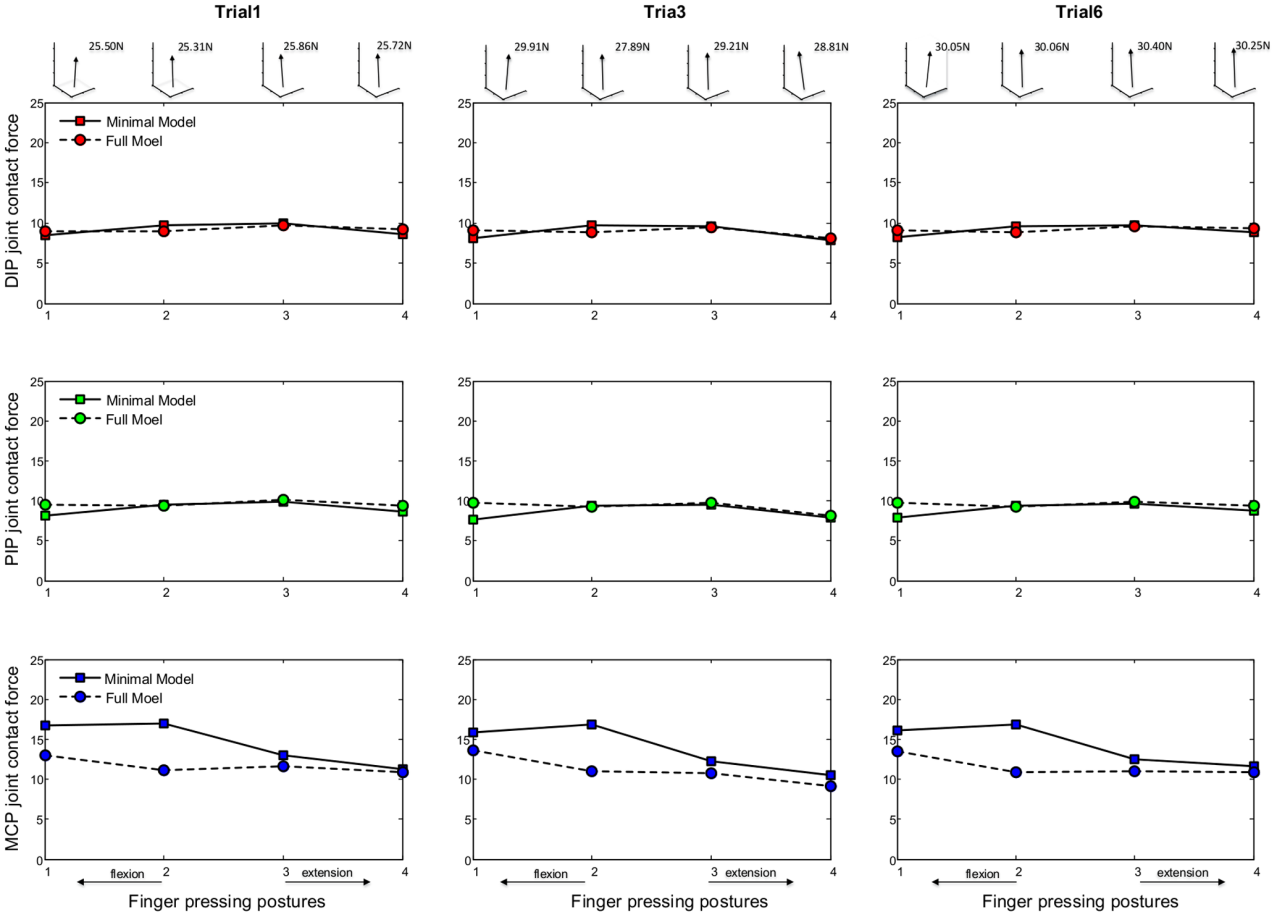
Bone-to-bone contact force calculation results. Calculated bone-to-bone contact forces (normalized by applied load) at the DIP, PIP and MCP joints obtained from both models for all finger postures. Based on measurement data from three typical trials (Trial 1, 3 and 6) for a representative subject (age: 25, weight: 75 kg, height: 1.72 m). The insets at the top show the measured 3D fingertip force vector for each posture.

Figure 6 compares the calculated bone-to-bone contact forces at the DIP, PIP and MCP joints obtained from the two finger models, for all four pressing postures, using measurement data from three typical trials (Trial 1, 3, 6) for a representative subject (age: 25, weight: 75 kg, height: 1.72 m). The corresponding numerical data are presented in Tables S1 and S2. The DIP and PIP joint contact forces calculated by both models, normalized by the applied fingertip load, are in the range 7.7–9.8 for all finger postures. The MCP joint contact forces calculated by both models are in the range 10.5–17.0 times the applied fingertip load. This agrees well with the estimated contact force ranges for the index finger interphalangeal and metacarpal joints from previous studies for isometric key pinching [32]. However, it should be noted that, in this study, maximum voluntary isometric pressing was conducted on a large force plate surface, which differs slightly from key pinching. It can be seen from Figure 6 that both models show the MCP joint contact force increasing with more flexed postures. This is in general agreement with the posture-dependent pattern of MCP joint contact force reported by Harding et al. [21]. Comparing the joint contact forces generated by the minimal model and the full model in Figure 6, it appears that including the extensor mechanism does not have a significant effect on the calculated DIP and PIP joint contact forces. However, an appreciable effect can be observed on the calculated MCP joint contact force, where the full model predicts much lower values, especially in more flexed finger postures.

**Figure 7:**
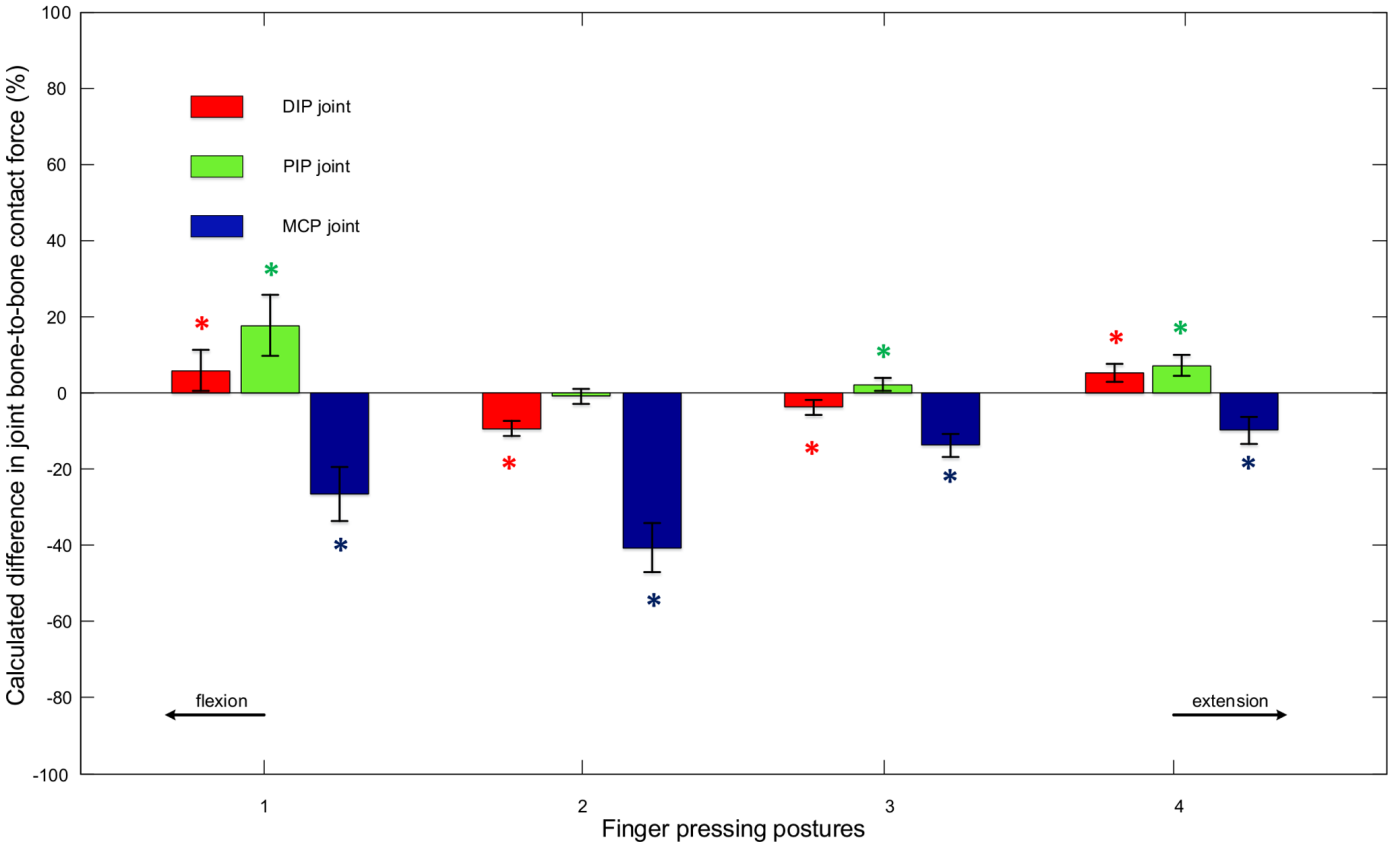
Percentage difference in bone-to-bone contact forces. The differences between the calculated bone-to-bone contact forces at the DIP, PIP and MCP joints obtained from the two models for all finger postures. The means and standard deviations were calculated across all trials and all subjects. A ‘*’ indicates a significant difference between the results of the two models.

Figure 7 shows the percentage differences between the contact forces calculated by the full model and those calculated by the minimal model for each pressing posture (means and standard deviations across all trials and all subjects). This further supports the observation that including the extensor mechanism has a limited effect on the calculated DIP and PIP joint contact forces. With the exception of the PIP joint in the most flexed posture, the mean differences for the DIP and PIP joints are within ±9% and there is no consistent trend as the finger becomes more flexed or more extended. However, there is a consistent negative difference for the calculated MCP joint contact force across all finger postures (i.e. the full model produces lower force estimates). This difference becomes more pronounced when the finger becomes more flexed. For the two most flexed postures, mean decreases of 27% and 41% in estimated MCP contact force are obtained when the extensor mechanism is included. If the full model is assumed to be realistic, this suggests that the tendon network of the extensor mechanism might help to moderate the joint contact loads at the MCP during isometric pressing and hence may reduce the risk of injury or osteoarthritis [33], [34].

In Figure 7, statistically significant differences (p<0.05) between models are labelled with a ‘*’, which indicates that the mean bone-to-bone contact forces calculated by the two models differ significantly. With the exception of the PIP joint in posture 2, the differences between the results from the two models are all statistically significant (i.e. the calculated joint contact forces are significantly different when the extensor mechanism is included). Statistically significant differences between postures for both models are presented in Tables 1 and 2.

**Table 1:** Statistical analysis of results from the Minimal Model.

	Muscle forces				Bone-to-bone contact forces		
	*F_PE_*	*F_PF_*	*F_RI_*	*F_UI_*	*F_DIP_*	*F_PIP_*	*F_MCP_*
**Posture1**	2.863±0.571^a^	5.150±0.802^a^	4.218±0.892^a^	4.121±0.832^a^	8.946±0.998^a^	8.641±0.917^a^	15.719±1.921^a^
**Posture2**	4.033±1.110^b^	8.293±1.987^b^	4.800±1.378^b^	7.437±3.502^b^	13.159±2.562^b^	12.879±2.557^b^	23.731±6.568^b^
**Posture3**	3.657±1.024^c^	8.817±1.442^c^	3.847±1.156^c^	7.059±2.562^b^	13.268±1.777^b^	13.032±1.731^b^	22.586±4.840^b^
**Posture4**	1.772±0.613^d^	7.565±1.793^d^	1.132±0.259^d^	2.375±0.649^c^	10.098±2.164^c^	10.092±2.166^c^	13.464±2.797^c^

Statistical analysis of the effect of finger posture on normalised muscle forces and joint bone-to-bone contact forces based on results from the Minimal Model.

Values are means ± s.e.m. for all trials and all subjects. Identical letters indicate posture groups within a column do not differ significantly from each other (p>0.05).

**Table 2:** Statistical analysis of results from the Full Model.

	Muscle forces				Bone-to-bone contact forces		
	*F_LE_*	*F_FDP_*	*F_RI_+F_LU_*	*F_UI_*	*F_DIP_*	*F_PIP_*	*F_MCP_*
**Posture1**	2.293±0.497^a^	4.992±0.932^a^	2.503±0.712^a^	2.287±0.788^a^	8.630±1.031^a^	9.028±1.348^a^	11.755±2.101^a^
**Posture2**	3.066±0.905^b^	7.999±2.308^b^	3.017±0.754^b^	3.066±1.598^b^	12.575±2.913^b^	13.693±3.502^b^	16.742±4.488^b^
**Posture3**	3.080±1.292^b^	8.671±1.498^c^	2.448±0.643^a^	3.075±1.287^b^	12.977±1.798^b^	14.025±2.173^b^	16.921±3.030^b^
**Posture4**	1.760±0.618^c^	7.446±1.728^d^	0.953±0.232^c^	1.294±0.446^c^	9.860±1.979^c^	10.064±2.041^c^	12.093±2.583^a^

Statistical analysis of the effect of finger posture on normalised muscle forces and joint bone-to-bone contact forces based on results from the Full Model.

Values are means ± s.e.m. for all trials and all subjects. Identical letters indicate posture groups within a column do not differ significantly from each other (p>0.05).

**Figure 8:**
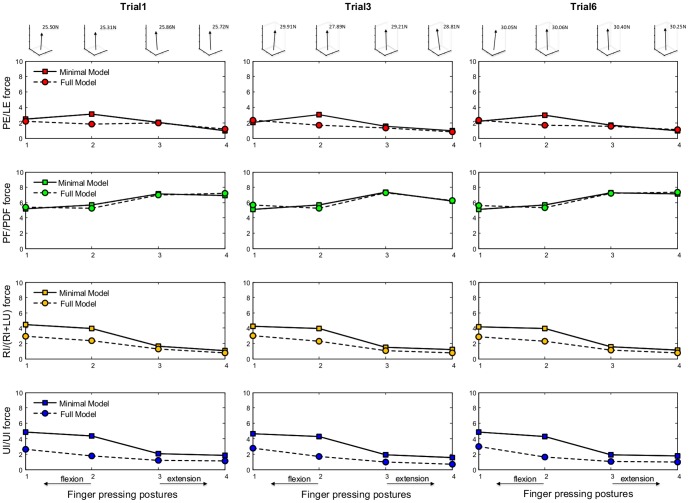
Muscle force calculation results. Calculated muscles forces (normalized by applied load) for the *PE* (*LE*), *PF* (*PDF*), *RI* (*RI+LU*) and *UI* muscles obtained from both models for all finger postures. Based on measurement data from three typical trials (Trial 1, 3 and 6) for a representative subject (age: 25, weight: 75 kg, height: 1.72 m). The insets at the top show the measured 3D fingertip force vector for each posture.

Figure 8 compares the calculated muscle forces obtained from the two finger models, for all four pressing postures, using measurement data from three typical trials (Trial 1, 3, 6) for a representative subject (age: 25, weight: 75 kg, height: 1.72 m). The corresponding numerical data are presented in Tables S1 and S2. The muscles from the two models are compared based on their anatomical functions, i.e. *PE* versus *LE* as extensors, *PF* versus *FDP* as flexors, *RI* versus *RI*+*LU* as lateral radial muscles and *UI* versus *UI* as lateral ulnar muscle. The range of muscle forces is approximately 1.2 to 7.0 times the applied fingertip load, which is in general agreement with the muscle force data reported in previous research on isometric pinching [30], [32], which is similar to pressing on a flat surface. It can be seen from Figure 8 that posture-dependent trends are present for the *PF* or *FDP*, *RI* or *RI*+*LU* and *UI* muscles. The intrinsic muscle forces (*RI* or *RI*+*LU* and *UI*) increase with more flexed postures. However, the extrinsic flexor muscle (*PF* or *FDP*) shows decreasing force when the finger becomes more flexed. This is in a good agreement with the posture-dependent trends of the *FDP* muscle reported in the study by Weightman and Amis [20]. If the full model is assumed to be realistic, then the results from the two models suggest that the extensor mechanism may have a significant effect on the *RI*+*LU* and *UI* muscles for all finger postures. The full model, including the extensor mechanism, predicts much lower *RI*+*LU* and *UI* muscle forces than those predicted by the minimal model without the extensor mechanism.

**Figure 9:**
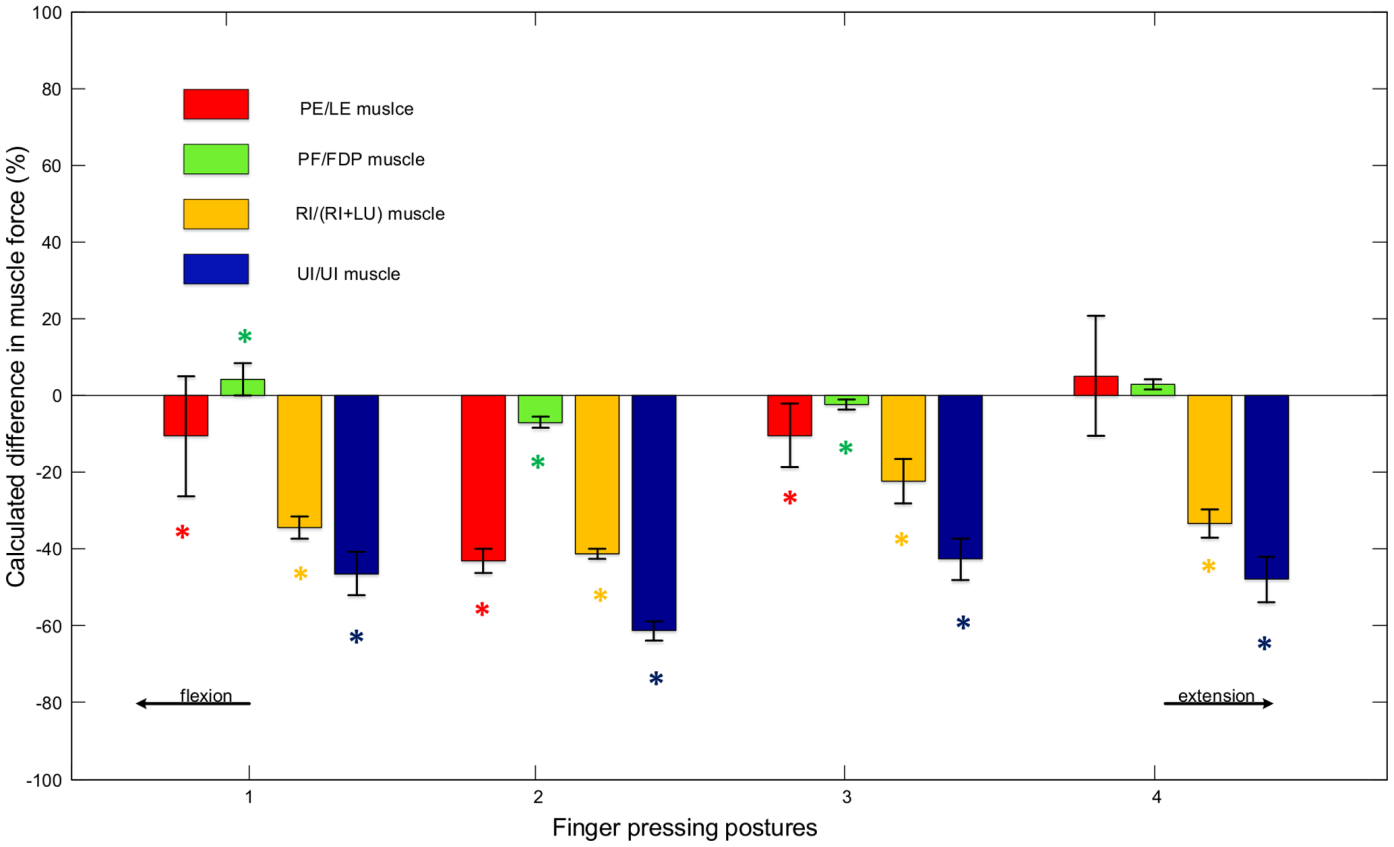
Percentage difference in muscle forces. The differences between the calculated muscle forces for the *PE* (*LE*), *PF* (*PDF*), *RI* (*RI+LU*) and UI muscles obtained from the two models for all finger postures. The means and standard deviations were calculated across all trials and all subjects. A ‘*’ indicates a significant difference between the results of the two models.

Figure 9 shows the percentage differences between the muscle forces calculated by the full model and those calculated by the minimal model for each pressing posture (means and standard deviations across all trials and all subjects). It can be seen that the *RI*+*LU* and *UI* muscle forces are notably reduced when the extensor mechanism is included. The differences increase in magnitude with more flexed pressing postures, reaching 34% to 61% at the two most flexed postures. The differences are very small for the *PF* or *FDP* muscle forces. This agrees with the results obtained by Li et al. [23] who used simple 2D models without extensor forces to investigate the effect of fingertip load on flexor forces during isometric pressing with a fully extended finger. It can be seen from Figure 9 that mixed results are obtained for the *PE* or *LE* muscles. At postures 1, 3 and 4 the differences are small but at posture 2 there is a large negative difference (43%). In conclusion, if the full model is assumed to be realistic, the muscle force results suggest that the extensor mechanism helps to reduce the intrinsic muscle forces (*RI, LU* and *UI*), and this may also be the case for the extrinsic extensor muscles at moderately flexed postures.

In Figure 9, statistically significant differences (p<0.05) between models are labelled with a ‘*’, which indicates that the mean muscle forces calculated by the two models differ significantly. With the exception of the *PE* (*LE*) and *PF* (*FDP*) in posture 4, the differences between the results from the two models are all statistically significant (i.e. the calculated muscle forces are significantly different when the extensor mechanism is included). Statistically significant differences between postures for both models are presented in Tables 1 and 2.

## Discussion and Conclusion

By combining experimental measurement with biomechanical modelling, this study has investigated the calculated effects of the finger extensor mechanism on the contact forces at the interphalangeal and metacarpal joints and also on the forces exerted by the intrinsic and extrinsic muscles. If the full model is assumed to be realistic, then the results from the two models suggest some biomechanical advantages that may be provided by the tendon network of the extensor mechanism. The estimated forces in the intrinsic muscles (interosseus group and lumbrical) are significantly reduced by 22% to 61% when the extensor mechanism is included, especially in more flexed postures. The estimated contact force at the MCP joint is decreased by 10% to 41%, with larger reductions in more flexed postures, when the extensor mechanism is included. These effects may help to reduce the risk of injury at the finger joints and may also help to moderate the muscular effort required of the finger's intrinsic muscles.

The apparent biomechanical advantages provided by the finger extensor mechanism may be a result of its distinctive anatomical arrangement. The extensor apparatus surrounding the MCP joint receives muscle forces from the lumbricals (*LU*) and interossei (*RI* and *UI*). The contraction of these intrinsic muscles produces PIP and DIP extension by transmitting tension through the tendon network of the extensor mechanism (see Figures 1 and 5). The extensor slip (*ES*) attaches to the intermediate phalanx, where tension transmitted through the tendon network due to the intrinsic muscles extends the PIP joint. The lateral bands (radial band *RB* and ulnar band *UB*) on the dorsal side of the PIP joint merge over the dorsum of the intermediate phalanx, forming the terminal extensor (*TE*) slip, and insert into the distal phalanx, where the intrinsic muscle contraction leads to extension of the DIP joint. The tension generated by the contraction of the intrinsic muscles at the DIP and PIP joints tends to increase the force at the *FDP* muscle which further contributes to the flexion moment at the MCP joint, and thereby reduces the force demand imposed on the intrinsic muscles and hence moderates the bone-to-bone contact force at the MCP joint.

The biomechanical models used in this study have some limitations. The extensor apparatus is modelled as a tendon network with the individual tendon components represented by lines. However, in reality the finger extensor mechanism is a complex assembly of multi-directional fibres of varying viscoelastic properties. Three-dimensional solid mechanics models (e.g. based on the finite-element method) would be needed to better represent this interconnected fibrous structure in the future. To calculate the muscle and tendon forces in the full model, a set of empirical equations obtained from previous studies (Equations 7–11) was used to resolve the static indeterminacy problem. A more sophisticated optimisation based method could be employed to improve the solution of this statically indeterminate system of muscles and tendons [35]–[38]. However, finding an appropriate optimisation criterion may be challenging.

## Supporting Information

Table S1 **Calculation results from the Minimal Model.** Force plate data and normalized calculation results from the Minimal Model for three typical trials (Trial 1, 3 and 6) with a representative subject (age: 25, weight: 75 kg, height: 1.72 m)
(DOCX)

Table S2 **Calculation results from the Full Model.** Force plate data and normalized calculation results from the Full Model for three typical trials (Trial 1, 3 and 6) with a representative subject (age: 25, weight: 75 kg, height: 1.72 m)
(DOCX)
