# Supplementary material for: Biomechanical Analysis of the Human Finger Extensor Mechanism during Isometric Pressing
Source: PLoS One. 2014 Apr 14;9(4):e94533. doi: 10.1371/journal.pone.0094533 (PMC3986208; doi:10.1371/journal.pone.0094533)
Supplement: Table S1 — Calculation results from the Minimal Model. Force plate data and normalized calculation results from the Minimal Model for three typical trials (Trial 1, 3 and 6) with a representative subject (age: 25, weight: 75 kg, height: 1.72 m) (DOCX) [file pone.0094533.s001.docx]

**Table S1.** **Calculation results from the Minimal Model.** Force plate data and normalized calculation results from the Minimal Model for three typical trials (Trial 1, 3 and 6) with a representative subject (age: 25, weight: 75kg, height: 1.72m)

|  |  | **Trial 1** | | | | **Trial 3** | | | | **Trial 6** | | | |
| --- | --- | --- | --- | --- | --- | --- | --- | --- | --- | --- | --- | --- | --- |
| **Postures** |  | **1** | **2** | **3** | **4** | **1** | **2** | **3** | **4** | **1** | **2** | **3** | **4** |
| **Force (N)** | $P_{x}$ | -0.95 | 2.05 | 4.18 | 4.16 | -2.72 | 2.21 | 2.16 | 7.16 | -1.81 | 2.12 | 3.11 | 4.12 |
|  | $P_{y}$ | 25.48 | 25.20 | 25.50 | 25.29 | 29.77 | 27.78 | 29.10 | 27.89 | 29.93 | 29.97 | 30.22 | 29.90 |
|  | $P_{z}$ | 0.90 | 1.18 | 0.98 | 2.10 | 1.01 | 1.15 | 1.11 | 1.02 | 1.97 | 1.02 | 1.20 | 2.00 |
| **Joint** |  |  |  |  |  |  |  |  |  |  |  |  |  |
| DIP | $F_{x\_DIP}$ | 2.73 | 4.11 | 6.98 | 6.57 | 2.55 | 4.07 | 6.68 | 6.04 | 2.62 | 4.05 | 6.79 | 6.73 |
|  | $F_{y\_DIP}$ | 8.08 | 8.83 | 7.15 | 5.58 | 7.65 | 8.77 | 6.92 | 5.13 | 7.80 | 8.73 | 7.01 | 5.71 |
|  | $F_{z\_DIP}$ | 0.04 | 0.04 | 0.04 | 0.08 | 0.03 | 0.04 | 0.03 | 0.03 | 0.07 | 0.03 | 0.03 | 0.07 |
| PIP | $F_{x\_PIP}$ | 6.14 | 7.32 | 8.11 | 6.65 | 5.75 | 7.25 | 7.77 | 6.11 | 5.89 | 7.21 | 7.89 | 6.81 |
|  | $F_{y\_PIP}$ | 5.43 | 6.02 | 5.60 | 5.46 | 5.14 | 5.98 | 5.42 | 5.02 | 5.24 | 5.95 | 5.49 | 5.60 |
|  | $F_{z\_PIP}$ | 0.04 | 0.04 | 0.04 | 0.08 | 0.03 | 0.04 | 0.03 | 0.03 | 0.07 | 0.03 | 0.03 | 0.07 |
| MCP | $F_{x\_MCP}$ | 16.67 | 16.98 | 12.8 | 9.60 | 15.82 | 16.86 | 12.16 | 8.91 | 16.13 | 16.80 | 12.41 | 9.84 |
|  | $F_{y\_MCP}$ | -0.79 | 0.60 | 1.81 | 5.97 | -0.74 | 0.59 | 1.78 | 5.53 | -0.76 | 0.59 | 1.79 | 6.11 |
|  | $F_{z\_MCP}$ | 0.005 | 0.004 | 0.001 | 0.01 | 0.004 | 0.004 | 0.001 | 0.004 | 0.008 | 0.004 | 0.001 | 0.008 |
| **Muscle** |  |  |  |  |  |  |  |  |  |  |  |  |  |
| *PE* | $F_{PE}$ | 2.46 | 3.14 | 2.06 | 0.96 | 2.03 | 3.06 | 1.52 | 0.94 | 2.19 | 3.02 | 1.70 | 0.97 |
| *PF* | $F_{PF}$ | 5.15 | 5.68 | 7.14 | 6.93 | 5.14 | 5.69 | 7.37 | 6.19 | 5.13 | 5.69 | 7.30 | 7.15 |
| *RI* | $F_{RI}$ | 4.43 | 3.93 | 1.60 | 1.02 | 4.27 | 3.94 | 1.49 | 1.17 | 4.17 | 3.94 | 1.55 | 1.12 |
| *UI* | $F_{UI}$ | 4.83 | 4.34 | 2.05 | 1.81 | 4.61 | 4.30 | 1.88 | 1.52 | 4.84 | 4.28 | 1.92 | 1.78 |
