# Supplementary material for: Biomechanical Analysis of the Human Finger Extensor Mechanism during Isometric Pressing
Source: PLoS One. 2014 Apr 14;9(4):e94533. doi: 10.1371/journal.pone.0094533 (PMC3986208; doi:10.1371/journal.pone.0094533)
Supplement: Table S2 — Calculation results from the Full Model. Force plate data and normalized calculation results from the Full Model for three typical trials (Trial 1, 3 and 6) with a representative subject (age: 25, weight: 75 kg, height: 1.72 m) (DOCX) [file pone.0094533.s002.docx]

**Table S2.** **Calculation results from the Full Model.** Force plate data and normalized calculation results from the Full Model for three typical trials (Trial 1, 3 and 6) with a representative subject (age: 25, weight: 75kg, height: 1.72m)

|  |  | **Trial 1** | | | | **Trial 3** | | | | **Trial 6** | | | |
| --- | --- | --- | --- | --- | --- | --- | --- | --- | --- | --- | --- | --- | --- |
| **Postures** |  | **1** | **2** | **3** | **4** | **1** | **2** | **3** | **4** | **1** | **2** | **3** | **4** |
| **Forces (N)** | $P_{x}$ | -0.95 | 2.05 | 4.18 | 4.16 | -2.72 | 2.21 | 2.16 | 7.16 | -1.81 | 2.12 | 3.11 | 4.12 |
|  | $P_{y}$ | 25.48 | 25.20 | 25.50 | 25.29 | 29.77 | 27.78 | 29.10 | 27.89 | 29.93 | 29.97 | 30.22 | 29.90 |
|  | $P_{z}$ | 0.90 | 1.18 | 0.98 | 2.10 | 1.01 | 1.15 | 1.11 | 1.02 | 1.97 | 1.02 | 1.20 | 2.00 |
| **Joint** |  |  |  |  |  |  |  |  |  |  |  |  |  |
| DIP | $F_{x\_DIP}$ | 2.90 | 3.74 | 6.78 | 7.05 | 2.91 | 3.71 | 6.60 | 6.14 | 2.92 | 3.70 | 6.65 | 7.11 |
|  | $F_{y\_DIP}$ | 8.53 | 8.10 | 6.96 | 5.95 | 8.62 | 8.04 | 6.84 | 5.20 | 8.63 | 8.01 | 6.87 | 6.00 |
|  | $F_{z\_DIP}$ | 0.04 | 0.04 | 0.04 | 0.08 | 0.03 | 0.04 | 0.03 | 0.03 | 0.07 | 0.03 | 0.03 | 0.07 |
| PIP | $F_{x\_PIP}$ | 8.49 | 8.37 | 9.05 | 7.94 | 8.61 | 8.27 | 8.57 | 6.82 | 8.63 | 8.21 | 8.71 | 7.95 |
|  | $F_{y\_PIP}$ | 4.26 | 4.18 | 4.53 | 5.01 | 4.54 | 4.18 | 4.66 | 4.43 | 4.46 | 4.17 | 4.61 | 5.09 |
|  | $F_{z\_PIP}$ | -0.02 | -0.21 | -0.08 | 0.05 | 0.05 | -0.22 | -0.06 | -0.04 | 0.09 | -0.22 | -0.07 | 0.02 |
| MCP | $F_{x\_MCP}$ | 13.00 | 11.11 | 11.47 | 9.16 | 13.57 | 10.93 | 10.57 | 7.68 | 13.52 | 10.83 | 10.84 | 9.07 |
|  | $F_{y\_MCP}$ | -0.17 | 1.07 | 1.98 | 5.93 | -0.22 | 1.07 | 1.96 | 5.05 | -0.22 | 1.07 | 1.96 | 5.91 |
|  | $F_{z\_MCP}$ | 0.07 | 0.09 | 0.05 | 0.05 | 0.05 | 0.09 | 0.04 | 0.04 | 0.06 | 0.09 | 0.04 | 0.05 |
| **Muscle** |  |  |  |  |  |  |  |  |  |  |  |  |  |
| *LE* | $F_{LE}$ | 2.21 | 1.82 | 1.98 | 1.22 | 2.31 | 1.72 | 1.31 | 0.81 | 2.32 | 1.66 | 1.52 | 1.08 |
| *FDP* | $F_{FDP}$ | 5.39 | 5.27 | 7.00 | 7.23 | 5.66 | 5.28 | 7.31 | 6.25 | 5.58 | 5.29 | 7.20 | 7.39 |
| *RI* | $F_{RI}$ | 1.28 | 0 | 0 | 0 | 1.73 | 0 | 0 | 0 | 1.53 | 0 | 0 | 0 |
| *LU* | $F_{LU}$ | 1.64 | 2.32 | 1.30 | 0.74 | 1.27 | 2.29 | 1.04 | 0.77 | 1.31 | 2.28 | 1.14 | 0.77 |
| *UI* | $F_{UI}$ | 2.62 | 1.73 | 1.19 | 1.11 | 2.79 | 1.67 | 0.98 | 0.68 | 2.95 | 1.64 | 1.02 | 0.99 |
| **Tendon** |  |  |  |  |  |  |  |  |  |  |  |  |  |
| *TE* | $F_{TE}$ | 2.70 | 2.73 | 1.92 | 1.27 | 2.55 | 2.65 | 1.46 | 1.01 | 2.63 | 2.62 | 1.61 | 1.21 |
| *RB* | $F_{RB}$ | 1.46 | 1.85 | 1.20 | 0.70 | 1.23 | 1.81 | 0.91 | 0.65 | 1.26 | 1.79 | 1.01 | 0.70 |
| *UB* | $F_{UB}$ | 1.24 | 0.88 | 0.73 | 0.57 | 1.31 | 0.84 | 0.55 | 0.36 | 1.37 | 0.82 | 0.59 | 0.51 |
| *ES* | $F_{ES}$ | 2.21 | 1.65 | 1.16 | 0.82 | 2.31 | 1.61 | 0.89 | 0.62 | 2.32 | 1.58 | 0.97 | 0.77 |
